# Enhancing Biosorbent Stability, Performance Efficiency, and Cost-effectiveness: A Ternary Magnetic Composite for Sequestration of Multiple Toxic Metals from Water

**DOI:** 10.1021/acs.langmuir.5c01248

**Published:** 2025-06-02

**Authors:** Aleksandër Peqini, Paul N. Diagboya, Seit Shallari, Ferdi Brahushi, Rolf-Alexander Düring

**Affiliations:** 1 Institute of Soil Science and Soil Conservation, Research Centre for BioSystems, Land Use and Nutrition (iFZ), 9175 Justus Liebig University Giessen, Heinrich-Buff-Ring 26, Giessen 35392, Germany; 2 Department of Environment and Natural Resources, Faculty of Agriculture and Environment, Agricultural University of Tirana, Tirana 1029, Albania; 3 Environmental fate of chemicals and remediation (EnFaCRe) laboratory, Department of Environmental Management and Toxicology, 664474 University of Delta, PMB 2090 Agbor, Nigeria

## Abstract

Innovative low-cost magneto-biochar-clay (MBC 1:2:1 and MBC 1:3:1) composite adsorbents formed by a one-step combination of magnetic nanoparticles (MNP), biochar (from grape cluster stalk), and feldspar clay were employed for Cd­(II), Cr­(VI), and Cu­(II) removal from simulated contaminated aqueous solution. The composites expressed higher cation exchange capacity and BET surface area compared to the feldspar clay, as well as characteristic biobased functional groups such as hydroxyls, carboxyls, and amides. The optimum removal efficiency was achieved at a 0.66 g/L solid-to-liquid ratio, and the equilibrium was attained at 720 min for all three ions. The adsorption process was via electrostatic interactions as well as adsorption within the pores (>90% of total adsorption). Surface functional groups involved in the adsorption process are −OH, −COO^–^, and −C–N. An increase of Cu­(II) concentration in solution enhanced Cr­(VI) removal efficiency by 86 and 79% on MBC 1:2:1 and MBC 1:3:1, respectively, while reducing Cd­(II) uptake by 65 and 52%, respectively. The equilibrium data was described by the Langmuir and Langmuir–Freundlich adsorption isotherm models. Higher temperature slightly enhanced Cd­(II) adsorption, while no temperature impact was observed for Cr­(VI) and Cu­(II) adsorption. The adsorbent reusability study confirmed that the removal efficiency for Cr­(VI) remained high after five cycles, while for Cd­(II) and Cu­(II) only during the first adsorption cycle. Thus, the MBC composite is a cost-effective and efficient adsorbent and, due to its magnetic properties, can easily be applied as a water treatment adsorbent for Cr­(VI) removal from water.

## Graphical abstract

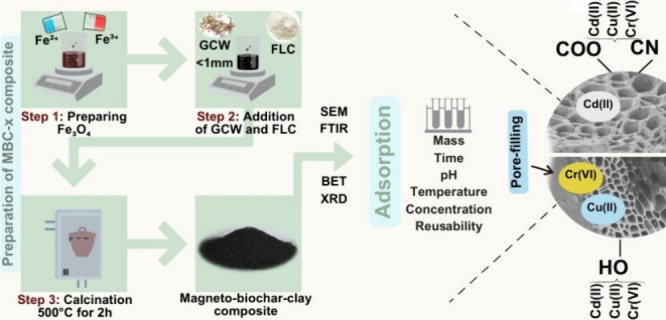

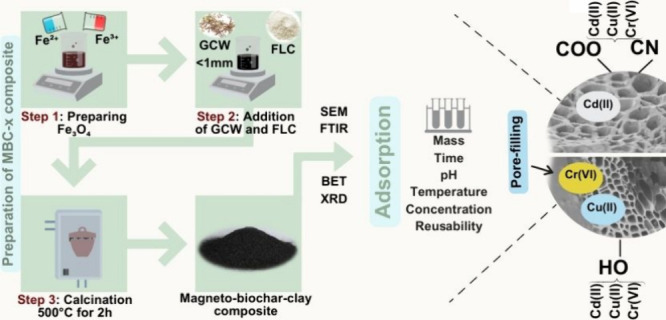

## 1 Introduction

In 2022, the World Health Organization reported that 3.5 billion people lack access to clean water, while 2.2 billion of these do not have access to clean potable water.^1^ Environmental pollutants, especially in water, are introduced via various anthropogenic activities, including agricultural, industrial, domestic, and other personal activities.^2–5^ A widely detected group of pollutants in the aqueous environment includes toxic metals. Toxic metals include Pb­(II), Hg­(II), Cd­(II), As­(III), Cr­(VI), and Cu­(II), and they are known to pose serious health risks to humans and other biota. For instance, Cd­(II) is reported to be toxic to the kidney, cause bone demineralization, and may impair lung function and increase the risk of lung cancer, while Cr­(VI) causes lung and skin cancer, and is neurotoxic.^6,7^ Cu­(II) is an essential element for biota but in excess amounts has been linked to the generation of highly reactive oxidative hydroxyl radicals, which damages the cells and DNA.^8^ Hence, it is vital to eliminate these toxic metals from water.

Several wastewater treatment techniques abound for contaminant removal from water, including chemical oxidation, precipitation, adsorption, biological techniques, and their combinations. Among these techniques, the simple, cost-efficient, environmentally friendly, and easy-to-handle adsorption is the most sought-after.^9,10^ There are several kinds of adsorbents such as synthetic nano- and mesoporous materials,^11,12^ clays,^13,14^ and biomass-based adsorbents.^15,16^ With appropriate treatment (e.g., calcination, pyrolysis) of this side-stream biomass and the availability of low-cost materials like clays, the adsorption technique is very promising. Application of low-cost agricultural biomass in this manner improves environmental aesthetics and reduces their adverse environmental effects, such as greenhouse gas emissions, leaching to soil and ground/surface water, potential fire sources, etc. Irrespective of the several advantages associated with biomass-derived materials, their application as the sole adsorbent suffers from several drawbacks, including low adsorption of contaminants, bleeding of the adsorbents, low stability, low pore size and surface area, and difficulty in adsorbent removal from treated water postadsorption.^17^ Hence, biomass adsorbents in synergistic combinations with other low-cost materials such as clays and nanoparticles have been studied.^18–21^ Most of the combinations have been aimed at enhancing the composite’s adsorption capacity and stability. Yet, another challenge is the separation of the adsorbents from large volumes of water postpurification. One proposed method of handling this is through magnetizing the adsorbent, thus eliminating the need for cumbersome and energy-demanding filtration postpurification.^22^ In the presence of a strong magnetic field, the magnetized adsorbents are pulled aside in a solid–liquid separation process, leaving the purified water on one side and the contaminant-filled adsorbent on the other.

In this study, grape cluster stem biowaste was obtained from a farm in Albania. Grape cluster waste runs in several tons yearly, and it should be put to alternative use to improve environmental aesthetics and reduce pollution. In addition, as Albania prepares to join the EU, there is a requirement for aligning its water policy with the Water Framework Directive (Directive 2000/60/EC), which requires the achievement of a good ecological and chemical status for all water bodies. The National Environment Agency of Albania reported in 2022 that the water quality of two out of six basins in Albania (Ishmi and Seman), evaluated through physicochemical parameters, resulted in a bad quality. Also, during a 1 year assessment in four different seasons of the Ishmi basin, Cd, Cr, and Cu were commonly detected (with 0.00015, 0.0043, and 0.00245 mg*/*L for Cd, Cr, and Cu, respectively (own data)). Thus, using a magnetic low-cost adsorbent prepared from local materials could help in reducing pollution and attaining the water standards set by the EU Directive on the protection of groundwater against pollution and deterioration (Directive 2006/118/EC) and Directive on environmental quality standards (Directive 2008/105/EC). Therefore, the study aimed to prepare cost-efficient magnetic adsorbents from feldspar clay and commonly available grape cluster biomass for the removal of three toxic metals (Cd­(II), Cr­(VI), and Cu­(II)) from water.

## 2 Materials and Methods

### 2.1 Materials and Pretreatment

Previously described pretreated feldspar clay (FLC)^13^ was employed in this study. Grape cluster stalk waste (GCW) was obtained from local vineyards in a central area of Albania. The GCW was washed with distilled water, dried (40 °C) to a constant weight, pulverized to fineness, sieved through a 1000 μm mesh sieve, and stored. Milli-Q (Purelab, UK) water at 18.2 MΩcm, Mettler Toledo scale (ME204), and pH meter (inoLab pH 7110) were used throughout the study. Analytical grade reagents were used throughout, including cadmium nitrate tetrahydrate (Merck, Germany), anhydrous copper chloride (Merck, Germany), potassium dichromate (Merck, Germany), iron­(III) chloride (Sigma-Aldrich, Germany), and iron­(II) sulfate heptahydrate (Carl Roth, Germany). Standard stock solutions (1000 mg/L each) of Cd­(II), Cr­(VI), and Cu­(II) were prepared in Milli-Q water from the above chemicals and stored, while the working solutions were prepared from these stocks.

### 2.2 Adsorbents and Magnetic Composite Preparations and Characterizations

The adsorbents used in this study were pristine Feldspar clay (FLC), biochar (BC), magnetic nanoparticles (MNP), and biochar-clay composites (MBC-*x*). The chemical coprecipitation of pure MNP was carried out by mixing FeCl_3_ (7.8 g) and FeSO_4_·7H_2_O (3.9 g) in 400 mL Milli-Q water at room temperature under continuous magnetic stirring,^18^ followed by dropwise addition of 2.0 M NaOH until the red/gray solution becomes black marking the completion of the MNP precipitation. The mixture was centrifuged for 5 min (2500 rpm), washed 6 times with Milli-Q, dried (40 °C), and weighed before storing it in an airtight container.

Pristine BC was obtained from the GCW by calcination of a known weight of GCW for 2 h in limited oxygen on a furnace ramped up at a temperature of ∼14 °C/min until 500 °C. The crucible was allowed to cool to room temperature in the furnace, sieved through to 1000 μm mesh sieve, washed 6 times with Milli-Q, dried (40 °C), and weighed before storing in an airtight container.

From the recorded weights of BC and MNP, the required masses to prepare MBC of ratios 1:2:1 and 1:3:1 were determined. From the adjusted weights, the MNP was prepared as described above but stopped before the centrifugation step, and then the adjusted GCW mass was added with shaking for 30 min at 200 rpm, followed by the addition of the adjusted FLC mass with further shaking for 2 h. The mixture was centrifuged (1500 rpm) for 10 min, dried overnight at 80 °C, and calcined in a crucible, as described above. The final composite materials were cooled, sieved, washed, weighed, and stored. The composites were labeled MBC-1:2:1 and MBC-1:3:1. Other variants of the MBC (with higher BC/FLC/MNP) were prepared, but since the ultimate goal of the composite(s) was to adsorb pollutants optimally while preserving the lowest possible magnetic property for easy removal of the adsorbent from water postadsorption, the two reported variants were the best in terms of better magnetic property and lower preparation cost.

The BC, FLC, MNP, and MBC composites were characterized by analytically determining their point of zero charge (pHpzc) using pH-drift method^23^ and cation exchange capacity (CEC) using the sodium saturation method,^24^ while the functional groups were determined using the Fourier transform infrared (FTIR) spectrometer (VERTEX70, Bruker Optics, Germany), crystallinity using X-ray diffractometer (XRD) (Empyrean spectrometer, Malvern Panalytical, Germany), specific surface area and porosity using a QUADRASORBevo analyzer (Quantachrome Instruments, USA), and surface morphology using a scanning electron microscope (SEM, Gemini SEM 560 Zeiss, Germany) with an energy-dispersive X-ray probe (EDX).

### 2.3 Sorption Experiments

Preliminary batch sorption experiments using monocontaminant and tricontaminant solutions were comparatively carried out using the BC, FLC, MNP, and MBC adsorbents. The results showed enhanced performances of the MBC 1:2:1 and MBC 1:3:1 composites; thus, these were employed in the detailed sorption study. Typically, each batch experiment employed 20 mg of mass of adsorbent in 30 mL of 5 mg/L contaminant solution (except where otherwise stated) at pH of 5.5 ± 0.1 (except for the effect of pH) in 50 mL plastic centrifuge tubes incubated for 1440 min (except where stated) at 200 rpm and 20 °C. The pH of the working solutions was adjusted with 0.2 M HCl/NaOH, where required. The effects of the following sorption parameters^25^ were examined: adsorbent mass (10–50 mg), time (1–1440 min), solution pH,^3–6^ contaminants concentration (1–20 mg/L), and experimental temperature (20, 30, and 40 °C). The reusability study was carried out using 20 mg of used adsorbent and solution metal concentrations of 0.5–10 mg/L. The adsorbed metals were desorbed by shaking in 30 mL of 0.2 M HCl twice (200 rpm for 1 h) and then in Milli-Q under the same conditions, before drying overnight and reusing. Five cycles of reusability were carried out. After each experiment, the samples were filtered with 0.45 μm PTFE filters, and metals in the filtrate were measured using an inductively coupled plasma–optical emission spectrometer (ICP-OES, Varian 720-ES).

### 2.4 Sorption Data Analysis

The amounts (mg/g) of cations adsorbed (*q*_*e*_) were calculated from eq 1, where *C*_*o*_, *C*_*e*_, *v,* and *m* are the initial and final concentrations (mg/L) of metal cations in solutions, experimental solution volume (mL), and mass (g) of adsorbent, respectively.

$$q_{e}=\left(C_{o}-C_{e}\right)v/m$$ 1

The nonlinear forms of the pseudo-first-order (PFOM)^26^ (eq 2), pseudo-second-order (PSOM) (eq 3), and the intraparticle diffusion (IPD) (eq 4)^27^ models were employed to explain the experimental rate data. The model quantities *q*_*e*_ and *q*_*t*_ are the amounts adsorbed (mg/g) at equilibrium and time *t*, and *k*_1_ (/min) and *k*_2_ (g/μg/min) are the rate constants of the PFOM and PSOM, respectively. The *K*_*i*_ (g/μg min^1/2^) is the rate parameter of the IPD control stage, and *C* (μg/g) is the estimated surface concentration of cations on the adsorbent surface.

$$q_{t}=q_{e}\left(1-e^{-k_{1}t}\right)$$ 2

$$q_{t}=\frac{q_{e}^{2}k_{2}t}{1+k_{2}q_{e}t}$$ 3

$$q_{t}=K_{i}t^{1/2}+C$$ 4

The equilibrium sorption data were fitted with nonlinear adsorption isotherm models of the Langmuir (eq 5), Freundlich (eq 6), and Langmuir–Freundlich (eq 7) type, where the models’ parameters *Q*_0_ or *q*_max_ (mg/g) are the maximum adsorption capacity per unit weight of adsorbent, *b* is the Langmuir constant, *K*_F_ is the Freundlich isotherm constant, 1/*n*_*F*_ is the Freundlich isotherm linearity parameter, and *K*_*lf*_ and *n* are a constant and the dimensionless exponent of Langmuir–Freundlich, respectively.

$$q_{e}=\frac{Q_{o}bC_{e}}{1+bC_{e}}$$ 5

$$q_{e}=K_{F}C_{e}^{1/n_{F}}$$ 6

$$q_{e}=\frac{q_{\max}K_{lf}C_{e}^{n}}{1+K_{lf}C_{e}^{n}}$$ 7

The equilibrium constant *K*_d_ (L/g) (eq 8) and thermodynamic parameters (enthalpy Δ*H*° (kJ/mol) (eq 9), entropy Δ*S*° (J/mol/K) (eq 9), and Gibbs free energy Δ*G*° (kJ/mol) (eq 10) were calculated using the equilibrium sorption data at 293.15, 303.15, and 313.15 K, where *R* is the universal gas constant. All fittings and models’ parameters were generated using the OriginPro2015 software (OriginLab Corporation, USA).

$$K_{d}=\frac{q_{e}}{C_{e}}$$ 8

$$\ln K_{d}=\frac{\Delta S^{{}^{\circ}}}{R}-\frac{\Delta H^{{}^{\circ}}}{RT}$$ 9

$$\Delta G^{{}^{\circ}}=-RT\ln K_{d}$$ 10

## 3 Results and Discussions

### 3.1 Characterization of Adsorbents

The main aim for the composite fabrication was to prepare an adsorbent that is economical, environmentally friendly, easy to separate from water post-treatment, and more effective in water treatment than any of the precursor materials. Hence, selected physical and chemical properties (Figures 1 and 2 and Table 1) of the precursor and composite adsorbents were analyzed and described here to prove that the composites meet these critical requirements. The results in Table 1 showed that the biochar exhibited a high or very alkaline pHpzc (Figure 1c), unlike the MNP and the feldspar, which expressed near-neutral and slightly acidic values, respectively. The final MBC composites exhibited pHpzc values that were closer to the major bulk material in the composite (biochar): the more biochar present, the higher the pHpzc value. The average composite pHpzc value was 9.3, and this pHpzc value was in line with those reported for clay-biochar composites.^15,18^ The pHpzc is one important parameter that indicates the adsorbent’s surface charge state in solution pH: below the pHpzc value, the average surface charge density is positive and the adsorbent would likely attract negatively charged ions, while it is negative above the pHpzc value and the surface would attract positive ions.^27^

**Figure 1:**
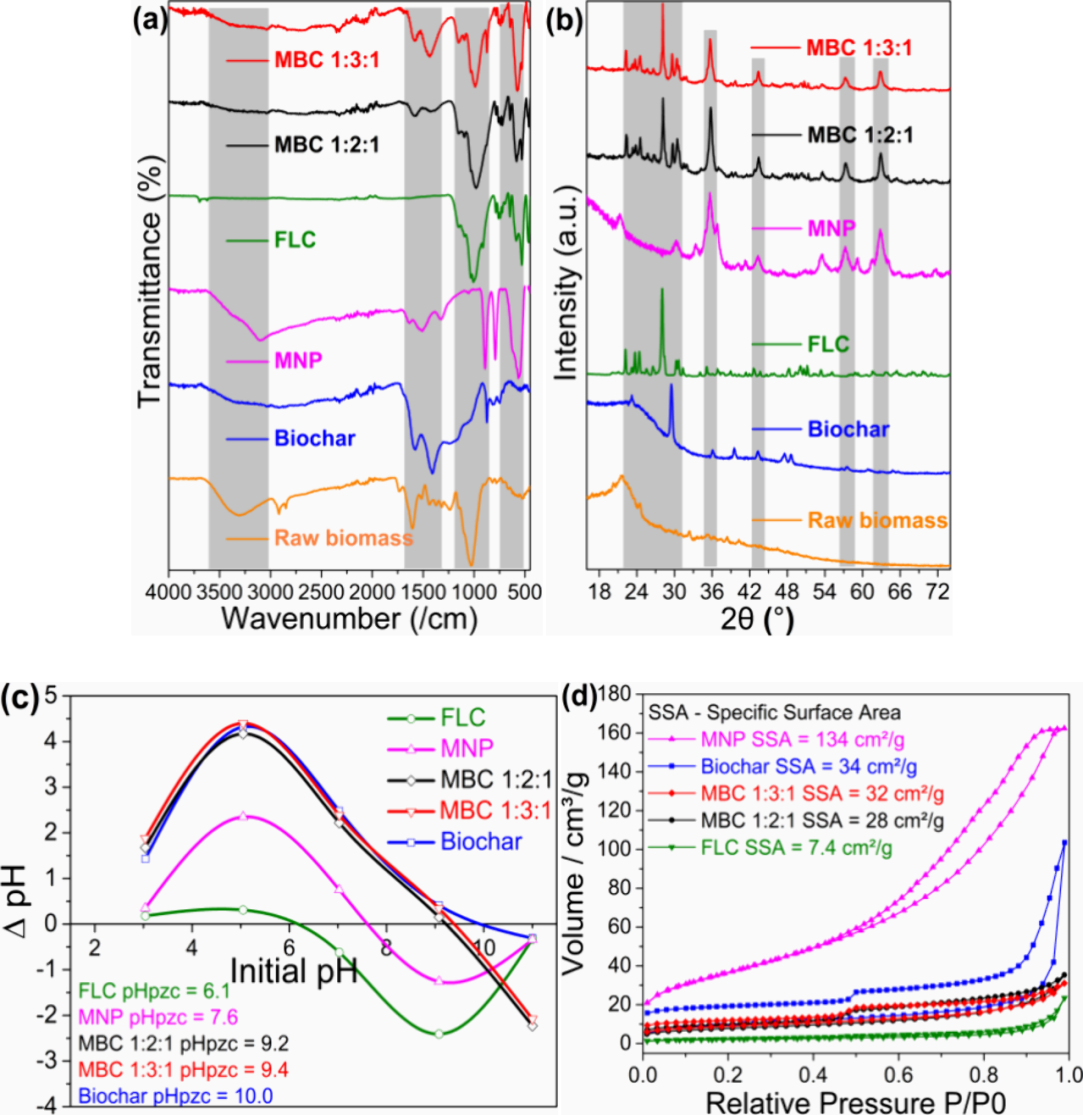
Characterization results of the biochar, FLC, MNP, MBC 1:2:1, and MBC 1:3:1 showing (a) FT-IR spectra peaks; (b) XRD diffractograms; (c) pHpzc; and (d) nitrogen adsorption/desorption isotherms (showing BET specific surface areas).

**Figure 2:**
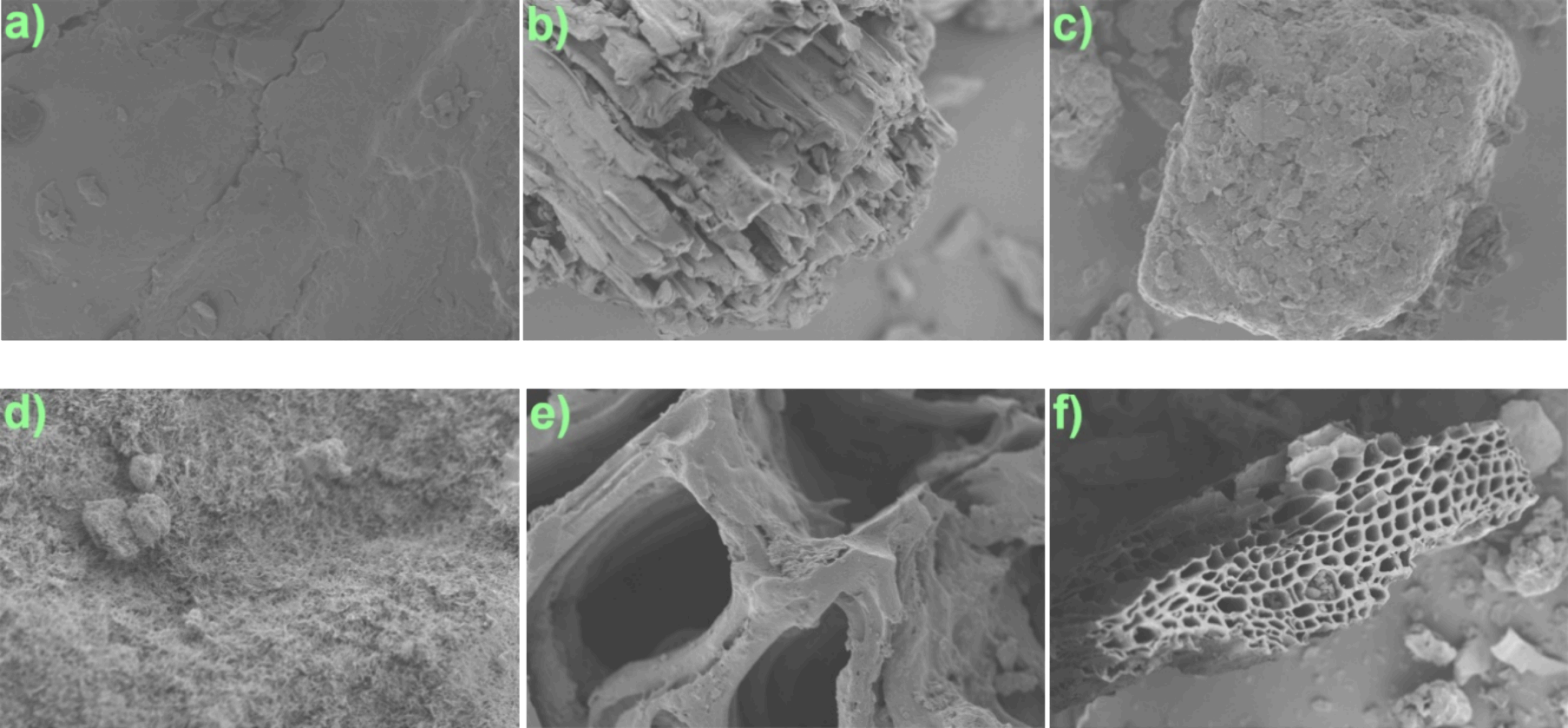
SEM electron micrographs of (a) biomass (magnification: 1 μm); (b) biochar (1 μm); (c) feldspar (1 μm); (d) magnetic nanoparticles (1 μm); (e) MBC 1:2:1 composite (1 μm); and (f) MBC 1:3:1 composite (10 μm).

**Table 1:** Physiochemical Characterization of Adsorbents

adsorbent	pHpzc	CEC (cmol/kg)	BET specific surface area (m^2^/g)
biochar	10.0	50.1	34
MNP	7.6	34	134
FLC	6.1	5.3	7.4
MBC 1:2:1	9.2	28.4	28
MBC 1:3:1	9.4	23.2	32

The values of the cation exchange capacity (CEC) (Table 1) of the composites showed an average decrease of ∼48 and 16%, respectively, when compared to the biochar and the MNP but higher than the feldspar. In addition to the low cost and higher stability of the composites, an average CEC of 24 mequiv/100g was considered effective. Similar to the CEC, the BET (Table 1 and Figure 1d) values were lower for the composites compared to those of the biochar and the MNP. The textural properties measured via nitrogen adsorption–desorption isotherms at 77 K (Figure 1 *d*) showed majorly IV isotherms with a sharp inflection usually occurring around relative pressures (*P*/*P*_0_) of 0.5–0.9.

Prediction and comparison of the major functional groups associated with the precursor and the final composites were done by monitoring the peaks between 4000 and 500/cm for biochar, MNP, FLC, MBC 1:2:1, and MBC 1:3:1 as shown in the FTIR spectra in Figure 1a. The peak around 1035/cm of the FLC and biomass were attributed to Si–O in-plane bending vibrations, while those at 730 and 525/cm of FLC were attributed to the Si–O–Si bridging bonds in SiO_2_ and Al–OH/Al–O deformations, respectively.^18^ These peaks were observed in the composites, though with slight shifts indicating the transfer of functional constituents from the precursors to the composites. Another major peak transferred to the composites is the characteristic magnetic Fe–O peak (around 560/cm) of the MNP; its presence is indicative of the magnetic property in the composites.^22^ The peaks between 1585 and 1420/cm, especially in the composites, are characteristic of C=O, C=C aromatic stretching rings, and amide-I groups, while those around 3200/cm of all materials (except FLC) were attributed to the presence of −OH groups.^26–29^

The degree of crystallinity measured through XRD (Figure 1b) showed the presence of characteristic FLC peaks for microcline, quartz, and feldspar minerals at 2θ of 22.0, 26.6, and 27.9°, respectively, with a partly triclinic feldspar mineral peak at 30.7°. Typical Fe_3_O_4_ MNP spinal structures were observed at 2θ values of 30.3, 35.5, 43.1, 57.1, and 62.7. Similar to the FTIR spectra, these crystal peaks in the FLC and MNP were observed in the composites, showing that there were no phase changes in the basic lattices of the FLC and MNP within the final composites.^18^

The morphologies of the precursor and final materials were examined via SEM (Figure 2a–f). The SEM of the raw biomass exhibited spongy amorphous surfaces (Figure 2a), which became less spongy with nonregular porous surfaces upon calcination to the biochar (Figure 2b), while the MNP exhibited spinal structures (Figure 2d). However, the morphologies of both MBC composites (Figure 2e,f) showed almost regular surfaces with shaped pores in the form of a honeycomb, confirming a successful preparation process.

Preliminary sorption experiments carried out to ascertain and compare sorption potentials of the precursor and composites showed enhanced removal efficiency for MBC 1:2:1 and MBC 1:3:1 of ∼370, 201, and 122% for Cd­(II), Cr­(VI), and Cu­(II), respectively, in comparison to the GCW, biochar (Figure S1a). In addition, the composites showed better removal efficiencies for Pb­(II) and for a model organic compound (terbuthylazine) of 99.7 and 99.0% for MBC 1:2:1, and 76.6 and 55.7% for MBC 1:3:1, respectively (Figure S1b). Thus, further experiments were conducted using MBC 1:2:1 and MBC 1:3:1 to obtain the optimized sorption parameters.

### 3.2 Optimization of Mass and pH, and Sorption Kinetics

For mass optimization of the composite adsorbents, a mass range of 10 up to 50 mg (0.33 and 1.66 g/L) was considered, using a 5 mg/L ternary contaminant solution, and the sorption trends are depicted in Figure 3a,b. Results showed that the Cd­(II) and Cu­(II) removal efficiency increased with a higher adsorbent mass. This was ascribed to the presence of numerous active exchangeable sites for these cations and the greater surface area of the adsorbents with increasing adsorbent mass.^30,31^ For the Cr­(VI) anion, however, the removal efficiency rose initially with an increase in adsorbent mass, but beyond the 20 mg mass, there was a downward deflection. A similar trend was reported for the coadsorption of Cd­(II) and Cr­(VI),^32^ and this may be attributed to the antagonistic effect between the two ions in solution during coadsorption. The results showed that the mass for optimum removal efficiency for all three cations was 20 mg; thus, this amount was selected for further experiments. At the 20 mg mass of MBC 1:2:1 and MBC 1:3:1, the optimum adsorption for Cd­(II) was 2.3 and 2.9 mg/g, respectively. These values for Cr­(VI) were 2.3 and 2.4 mg/g, while those for Cu­(II) were 7.6 and 8.3 mg/g, accordingly.

**Figure 3:**
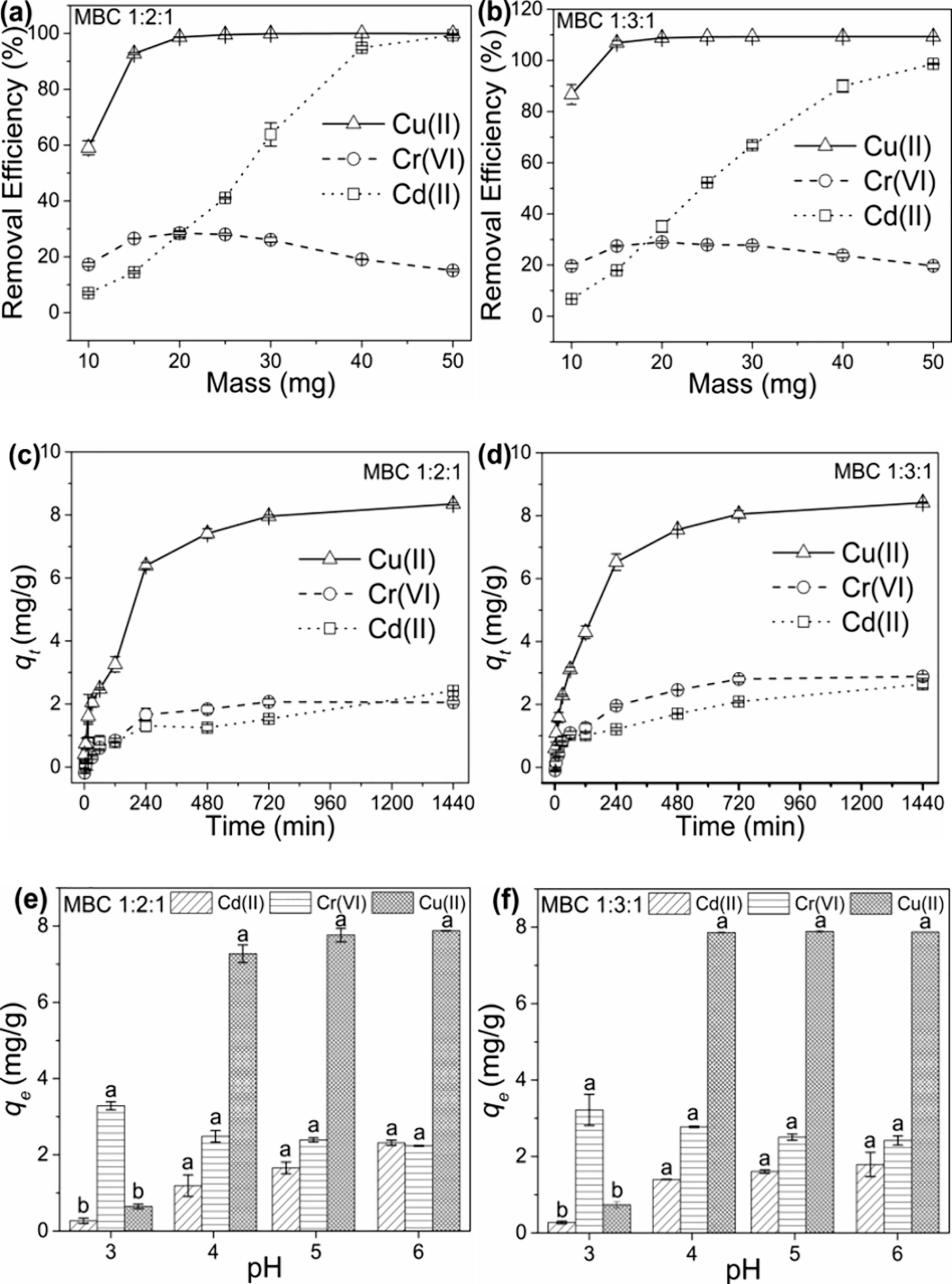
Adsorption trends at varying masses of (a) MBC 1:2:1 and (b) MBC 1:3:1; adsorption rate trend on (c) MBC 1:2:1 and (d) MBC 1:3:1; and adsorption trend at varying solution pH for (e) MBC 1:2:1 and (f) MBC 1:3:1.

The sorption rate trends for Cd­(II), Cr­(VI), and Cu­(II) ions on the composites are presented in Figure 3c,d. Both composites showed fast ion uptake over the first 240 min, and this may be attributed to the presence of plenty of empty active adsorption sites on the adsorbent surfaces.^33^ This initial fast adsorption was followed by a slower increase in trend up to 720 min, which was ascribed to the filling of the remaining composite adsorption sites and intraparticle diffusion of cations within the composites.^34^ Though a significant portion of the adsorption of the contaminants (≥85%) occurred before 240 min, equilibrium was attained for both composites at 720 min. Therefore, further tests were carried out at 720 min. The mass and rate experiments showed that the affinity of the composites for Cu­(II) ions was far higher than for Cd­(II) and Cr­(VI); the general trend was Cu­(II) > Cd­(II) ≥ Cr­(VI).

Another important parameter in evaluating the efficiency of a new adsorbent is its response to pH changes. This is vital because it directly affects both the metal ions’ speciation and composite surface charge.^35^ Therefore, the influence of solution pH on the adsorption process by the MBC composites was evaluated and is presented in Figure 3e,f. The significantly low (*p* < 0.05) sorption for Cd­(II) and Cu­(II) observed in the acidic conditions (pH 3) may be ascribed to partial protonation of the functional groups, and the competition between H^+^ (or H_3_O^+^) and metal, both cations for the negatively charged adsorption sites on the MBC composites.^33^ Thus, by increasing the solution pH, the proton concentration decreases and the adsorption sites become progressively negative, resulting in a lower competition between protons and cations and more electrostatic interactions with Cd­(II) and Cu­(II) on both composites.^36^ The optimum pH for adsorption of Cd­(II) and Cu­(II) was at pH 6. However, for Cr­(VI) anions, which exist as HCrO_4_^–^ at low pH,^37^ the adsorption was higher at pH 3 as a result of electrostatic attraction between HCrO_4_^–^ and positively charged adsorbent surfaces, especially at pH 3.^11^ Thus, the optimum adsorption of Cr­(VI) occurred at pH 3.

In evaluating the removal kinetics of Cd­(II), Cr­(VI), and Cu­(II) by MBC 1:2:1 and MBC 1:3:1, the PFOM, PSOM, and IPD kinetic models were employed. The fitting plots and model parameters are given in Figure S2a–f and in Table 2, respectively. In general, by comparing the PFOM and PSOM correlation coefficients (*r* ^2^) and chi-square (χ^2^), the PSOM exhibited better trends with values closer to unity and smaller, respectively. These show that the model fits the rate data and indicates that the adsorption process was controlled by the valence electron exchange between surface adsorption sites and metal ions, possibly leading to electrostatic interactions.^22,38,39^ The IPD model was evaluated to gain insight into the particulate nature (surface-to-pore relationship) of the adsorption process on the MBC composites. The *C* (mg/g) parameter of this model, which is an indication of the extent of surface adsorption, was lower than the experimental values (<10% of total adsorption) for both composites, suggesting that metal ions adsorption within the pores of the composites played some role in the adsorption process.

**Table 2:** Kinetic Model Parameters for MBC 1:2:1 and MBC 1:3:1 Adsorption

kinetic model	parameter	Cd(II) MBC 1:2:1	Cd(II) MBC 1:3:1	Cr(VI) MBC 1:2:1	Cr(VI) MBC 1:3:1	Cu(II) MBC 1:2:1	Cu(II) MBC 1:3:1
PFO	*q*_e_ (mg g^–1^)	1.831	2.249	2.049	2.760	8.141	8.031
	*k*_1_ (min^–1^)	0.005	0.004	0.005	0.006	0.005	0.007
	*r* ^2^	0.701	0.806	0.946	0.962	0.965	0.967
	χ^2^	0.145	0.129	0.038	0.044	0.334	0.299
PSO	*q*_e_ (mg g^–1^)	2.120	2.482	2.389	3.162	9.454	9.103
	*k*_2_ (g mg^–1^ min^–1^)	0.003	0.003	0.002	0.002	0.0007	0.001
	*r* ^2^	0.779	0.875	0.942	0.980	0.968	0.981
	χ^2^	0.107	0.083	0.041	0.023	0.308	0.174
IPD	*C* (mg g^–1^)	0.220	0.251	0.124	0.231	0.816	1.198
	*k*_i_ (g g^–1^ min^1/2^)	0.055	0.065	0.064	0.085	0.234	0.236
	*r* ^2^	0.910	0.953	0.830	0.891	0.883	0.875
	χ^2^	0.043	0.031	0.122	0.130	1.135	1.150
experimental	mg/g	2.413	2.636	2.048	2.888	8.348	8.414

### 3.3 Effect of Initial Concentration and Coexisting Metal Ions

The effect of initial concentration results (Figure 4a,b) showed that increasing initial Cu­(II) concentration in solution enhanced Cr­(VI) removal efficiency by 86 and 79% on MBC 1:2:1 and MBC 1:3:1, respectively, while reducing Cd­(II) uptake by 65 and 52%, respectively. Thus, in a ternary metal solution of Cr­(VI), Cu­(II), and Cd­(II), increasing the Cu­(II) concentration would enhance Cr­(VI) uptake while reducing the adsorption of Cd­(II). Interestingly, it was also observed that as the concentration increased, the Cr­(VI) removal attained ∼100% efficiency, while the Cu­(II) efficiency decreased from ∼100 to ∼90%. This may be due to the Cu­(II) adsorption site occupation (displacement of adsorbed ions) by Cr­(VI) as concentrations increased. This suggests that increasing the adsorbed positive charge on the adsorbent surface leads to higher electrostatic interaction with the Cr­(VI) anion, possibly in a multilayer. A similar trend has been reported earlier.^40^

**Figure 4:**
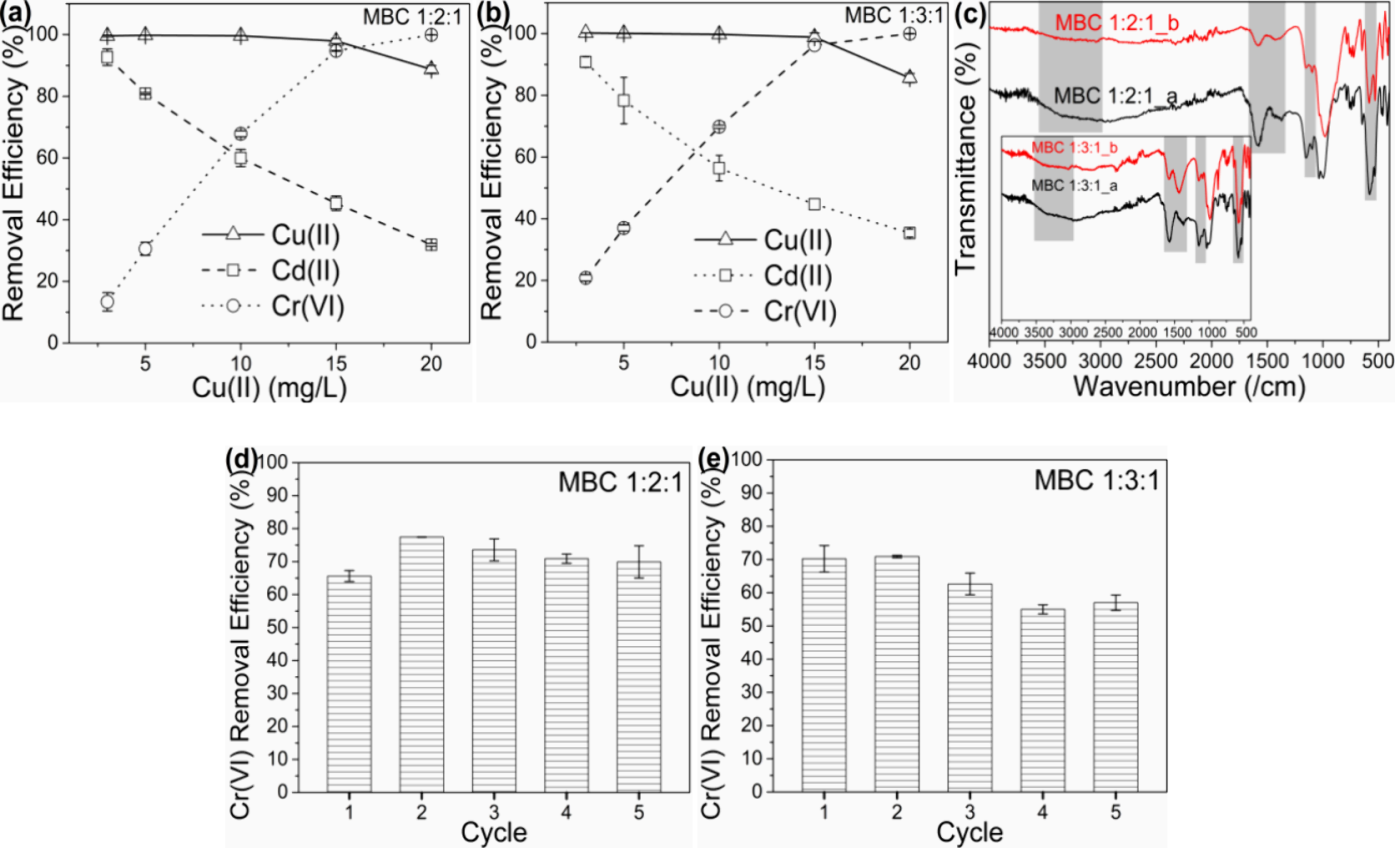
(a) Effect of the initial concentration of Cu­(II) on Cd­(II) and Cr­(VI) removal efficiency on MBC 1:2:1; (b) on MBC 1:3:1 [adsorbent mass: 20 mg in 30 mL solution using 1 mg/L Cd­(II), 3 mg/L Cr­(VI), and 3–20 mg/L of Cu­(II), pH = 5.4 at 200 rpm]; (c) pre- and postadsorption FTIR spectra of the composites; (d) reusability of Cr­(VI) on MBC 1:2:1; and (e) on MBC 1:3:1.

The adsorption mechanism in a multicomponent system could be explained based on ionic properties (Table S1). The higher adsorption of Cu­(II) may be attributed to its smaller hydrated radius and higher electronegativity (highest standard reduction potential) compared to Cd­(II) and Cr­(VI); this results in easier access into the pores and greater electrostatic interactions with the composite adsorption sites.^21,41^ To determine the interactions in a ternary system, the relative metal *i* adsorption *R*_*i*_(%) (eq 11)^32^ was employed, as given in Table S2.

$$R_{i}=\frac{\mathrm{metal}\;i\;\mathrm{adsorption}\;\mathrm{capacity}\;\mathrm{with}\;\mathrm{the}\;\mathrm{coexistence}\;\mathrm{of}\;\mathrm{metal}\;j\;\mathrm{and}\;k}{\mathrm{metal}\;i\;\mathrm{adsorption}\;\mathrm{capacity}\;\mathrm{without}\;\mathrm{coexistence}\;\mathrm{of}\;\mathrm{metal}\;j\;\mathrm{and}\;k}\times 100$$ 11

Based on this parameter, if *R*_*i*_ > 100%, then the interactive effect of mixed ions is synergistic, while *R*_*i*_ < 100% means there were antagonistic reactions. However, *R*_*i*_ = 100% shows noninteractive behavior. From the results (Table S2), Cd­(II) exhibited a synergistic and antagonistic effect for MBC 1:2:1 and MBC 1:3:1, respectively, while Cu­(II) and Cr­(VI) exhibited synergistic effects for both composites.

The effect of the metal adsorption on the FTIR spectra was examined and is depicted in Figure 4c. The peaks at around 3200/cm, which were attributed to the −OH groups, showed enhanced vibration after adsorption, indicating strong interaction with the metal ions.^21,42^ The −OH groups might have served as electron donors for Cr­(VI) reduction to Cr­(III) while being oxidized to carboxyl groups.^43^ This was confirmed by the corresponding carboxyl peak increase at 1585/cm after adsorption. Another source of electrons for Cr­(VI) reduction might have been iron oxide (Fe°) ions present in the composites.^37,42,44^ Amide-I groups at around 1420/cm exhibited higher vibrations and shifted after adsorption, indicating that these groups were active in the metal ion adsorption process.

### 3.4 Effect of Temperature on Sorption and Adsorption Isotherm Modeling

The adsorption of Cd­(II), Cr­(VI), and Cu­(II) on the MBC composites at fixed solution concentrations of Cd­(II) (1 mg/L) and Cr­(VI) (1 mg/L), and at varying concentrations (3–20 mg/L) of Cu­(II) at three different temperatures (20–40 °C), are given in Figure S4a–f. The sorption trends showed that with increasing Cu­(II) concentration, Cd­(II) sorption decreased (Figure S4a,b), especially at low temperature (20 °C). The slightly higher Cd­(II) and Cu­(II) (only on MBC 1:3:1) uptake at higher temperatures may be attributed to the endothermic nature of the process. On the other hand, no significant change in the sorption of Cr­(VI) was observed as the temperature was varied (Figure S4c–f). The calculated thermodynamic parameters showed that Δ*G*° < 0, Δ*S*° > 0, and Δ*H*° > 0 for MBC 1:3:1 (Table 3), indicating a spontaneous and endothermic process. The negative Δ*G*° values implied that the adsorption process is spontaneous. Moreover, the Δ*G*° decreases with temperature increase, suggesting that higher temperature conditions favor the adsorption process, thus enhancing the spontaneity of the process.^45^ This was supported by the positive Δ*H*° (10.10 kJ/mol) value, which indicated an endothermic process. Additionally, the positive Δ*S*° value (47.95 J/mol/K) signaled an increase in randomness at the solid-solution interface as the process proceeded toward equilibrium.

**Table 3:** Sorption Thermodynamic Parameters for Cu­(II) on MBC 1:3:1

temperature	Δ*G*° (kJ/mol)	Δ*H*°(kJ/mol)	Δ*S*° (J/mol/K)
293.15 K (20 °C)	–3.95	10.10	47.95
303.15 K (30 °C)	–4.41		
313.15 K (40 °C)	–4.91		

Due to the higher affinity of the composites for Cu­(II), the Cu­(II) adsorption isotherm models were evaluated at fixed Cd­(II) and Cr­(VI) concentrations, 1 and 3 mg/L, respectively, at three different temperatures (20, 30, and 40 °C). The employed model parameters (Langmuir, Freundlich, and Langmuir–Freundlich) are shown in Table 4 (Figure S3a–f). The *r* ^2^ (≥0.87) and χ^2^ (≤12.06) values suggested that the Langmuir adsorption isotherm model fit the data better than the Freundlich model, and this is an indication that the Cu­(II) adsorption process occurred on similar adsorption sites and in a monolayer. The relatively good fits of the Langmuir–Freundlich model (*r* ^2^ ≥ 0.87; χ^2^ ≤ 12.28) corroborate the idea that the adsorption occurred on heterogeneous adsorption sites but with a similar affinity or energy for Cu­(II).

**Table 4:** Cu­(II) Adsorption Model Parameters

		Cu(II) MBC 1:2:1			Cu(II) MBC 1:3:1		
adsorption isotherm model	parameter	20 °C	30 °C	40 °C	20 °C	30 °C	40 °C
Langmuir	*Q*_o_ (mg/g)	27.88	26.36	28.02	24.95	28.87	29.26
	β	26.12	46.33	30.55	142.16	33.07	22.58
	*r* ^2^	0.95	0.94	0.93	0.87	0.95	0.92
	χ^2^	4.29	5.55	6.87	12.06	4.43	8.26
Freundlich	1/*n*	0.22	0.19	0.20	0.15	0.18	0.20
	*K*_F_	24.97	24.82	24.99	24.93	24.82	25.07
	*r* ^2^	0.81	0.91	0.76	0.84	0.64	0.69
	χ^2^	19.25	9.01	24.46	15.70	35.55	31.43
Langmuir–Freundlich	*q*_max_ (mg/g)	27.95	30.68	28.63	29.50	27.41	28.97
	1/*n*	0.98	0.56	0.90	0.52	1.41	1.05
	*K*_LF_	24.83	23.54	20.05	8.73	180.8	28.10
	*r* ^2^	0.93	0.99	0.90	0.94	0.96	0.88
	χ^2^	6.43	0.42	10.04	2.36	3.87	12.28

### 3.5 Reusability and Cost Implications

The reusability of the MBC composite, an economic indicator of its applicability, was performed, and the results of five cycles are shown in Figure 4d,e. The average removal efficiency of the composites for Cd­(II) and Cu­(II) in the first cycle was ∼20 and 87%, respectively. In subsequent adsorption cycles, no significant removal of these cations was observed; thus, the reuse of these composites for both cations was not possible. This may be ascribed to the strong attachments of these cations to the composite’s adsorption in the first cycle. However, Cr­(VI) adsorption of the composites exhibited relatively constant efficiencies through the consecutive cycles (Figure 4d,e) with slight decreases (<10%) in efficiency for the MBC 1:3:1 toward the fifth cycle. The recorded Cr­(VI) removal efficiencies in the fifth cycle were 70 and 57% for MBC 1:2:1 and MBC 1:3:1, respectively. The subsequent inability of the composites to adsorb Cd­(II) and Cu­(II) but enhanced Cr­(VI) adsorption is an indication of cooperative adsorption after the first cycle. Thus, the used composites may be reusable for Cr­(VI) removal from water.

The cost implication of applying the MBC composites was estimated as reported by others for similar adsorbents^46,47^ by considering the cost of all precursor materials, treatments (energy and chemicals), and post-treatments (washing, drying, packaging, and transport) based on the reported calculation. The estimated costs per kilogram are ∼5.54 United States $ (Table S3). Considering the reusability of these adsorbents, this is far cheaper than several reported low-cost adsorbents.^46–49^

## 4 Conclusions

The starting individual materials of grape cluster biomass, feldspar clay, and Fe_3_O_4_ were successfully combined into ternary magneto-biochar-clay composites in MBC 1:2:1 and MBC 1:3:1. This was confirmed by following higher cation exchange capacity and BET surface area values compared to feldspar clay. Similarly, characteristic properties of the individual starting materials, such as unique infrared and XRD peaks as well as the basic crystalline structure of the feldspar, were transferred to the MBC composite. These adsorbents were applied in simultaneous aqueous multimetal ions (Cd­(II), Cr­(VI), and Cu­(II)) adsorption. Preliminary data showed enhanced MBC adsorption efficiency in comparison to the individual adsorbents. The average enhanced efficiencies of both composites for Cd­(II), Cr­(VI), and Cu­(II) were ∼370, 201, and 122%, respectively. The adsorption processes were mainly controlled by electrostatic interactions and adsorption on the pores. The surface functional groups involved in the process are −OH, −COO^–^, and −C–N. The Cu­(II) ion was the most adsorbed, and equilibrium was achieved in 720 min for all three metal ions. The optimal pH of adsorption for Cd­(II) and Cu­(II) was recorded at solution pH 6, while at pH 3 for Cr­(VI). Cooperative adsorption was observed between Cu­(II) and Cr­(VI) in a multimetal solution, while Cd­(II) adsorption decreased. Generally, the process was spontaneous but endothermic and was described by Langmuir and Langmuir–Freundlich adsorption isotherm models. The composites showed good removal efficiency for Cd­(II) and Cu­(II) during the first adsorption cycle and for Cr­(VI) even after five consecutive cycles; thus, the composite is a low-cost, easy-to-handle, feasible, and sustainable adsorbent for application in water treatment.

## Supplementary Material



## Notes

Removal efficiency of metal ions and terbuthylazine on MBC 1:2:1, MBC 1:3:1, biochar, FLC, and MNP; kinetics modeling of rate data; isotherm model fittings for equilibrium data; effect of temperature on adsorption; ionic properties of Cd­(II), Cr­(VI) and Cu­(II); interactions of metal ions in ternary systems; cost analysis of MBC 1:2:1/MBC 1:3:1 production (PDF)
