# Supplementary material for: Enhancing Biosorbent Stability, Performance Efficiency, and Cost-effectiveness: A Ternary Magnetic Composite for Sequestration of Multiple Toxic Metals from Water
Source: Langmuir. 2025 Jun 2;41(23):15022–30. doi: 10.1021/acs.langmuir.5c01248 (PMC12177914; doi:10.1021/acs.langmuir.5c01248)
Supplement: Supplementary file 1 [file la5c01248_si_001.pdf]

**SUPPLEMENTARY INFORMATION**

**Enhancing biosorbent stability, performance efficiency, and cost-effectiveness: a ternary magnetic composite for sequestration of multiple toxic metals from water**

Aleksandër Peqini[1, 2*], Paul N. Diagboya[3], Seit Shallari[2], Ferdi Brahushi[2], Rolf-Alexander Düring[1]

[1]Institute of Soil Science and Soil Conservation, Research Centre for BioSystems, Land Use and Nutrition (iFZ), Justus Liebig University Giessen, Heinrich-Buff-Ring 26, 35392 Giessen, Germany

[2]Department of Environment and Natural Resources, Faculty of Agriculture and Environment, Agricultural University of Tirana, 1029 Tirana, Albania

[3]Environmental fate of chemicals and remediation (EnFaCRe) laboratory, Department of Environmental Management and Toxicology, University of Delta, Agbor, Nigeria

[*]Corresponding author

Email: aleksander.peqini@umwelt.uni-giessen.de

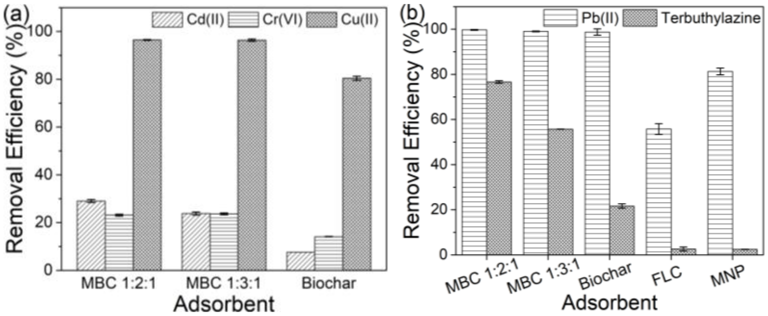


SI Fig. 1 Removal efficiency of (a) Cd(II), Cr(VI), and Cu(II) on MBC 1:2:1, MBC 1:3:1, and biochar; and (b) Pb(II) and terbuthylazine on MBC 1:2:1, MBC 1:3:1, biochar, FLC, and MNP

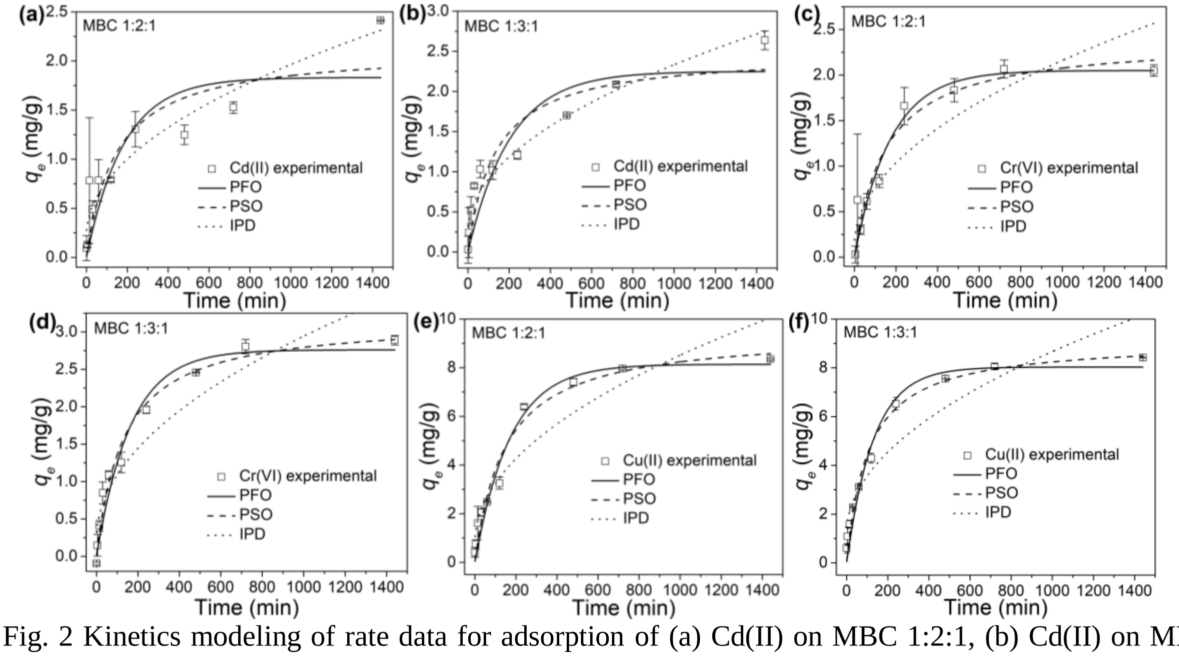


SI Fig. 2 Kinetics modeling of rate data for adsorption of (a) Cd(II) on MBC 1:2:1, (b) Cd(II) on MBC 1:3:1; (c) Cr(VI) on MBC 1:2:1 (d) Cr(VI) on MBC 1:3:1; (e) Cu(II) on MBC 1:2:1, (f) Cu(II) on MBC 1:3:1

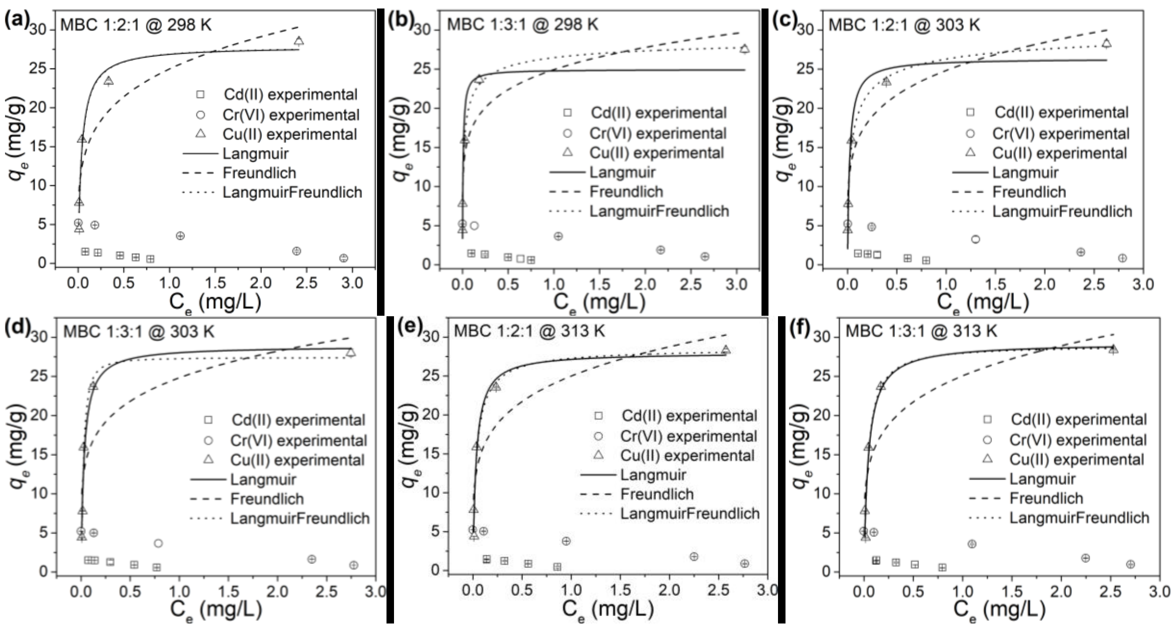


SI Fig. 3 Isotherm model fittings for Cu(II) on (a) MBC 1:2:1, (b) MBC 1:3:1 at 20 °C, (c) MBC 1:2:1, (d) MBC 1:3:1 at 30 °C, (e) MBC 1:2:1, (f) MBC 1:3:1 at 40 °C

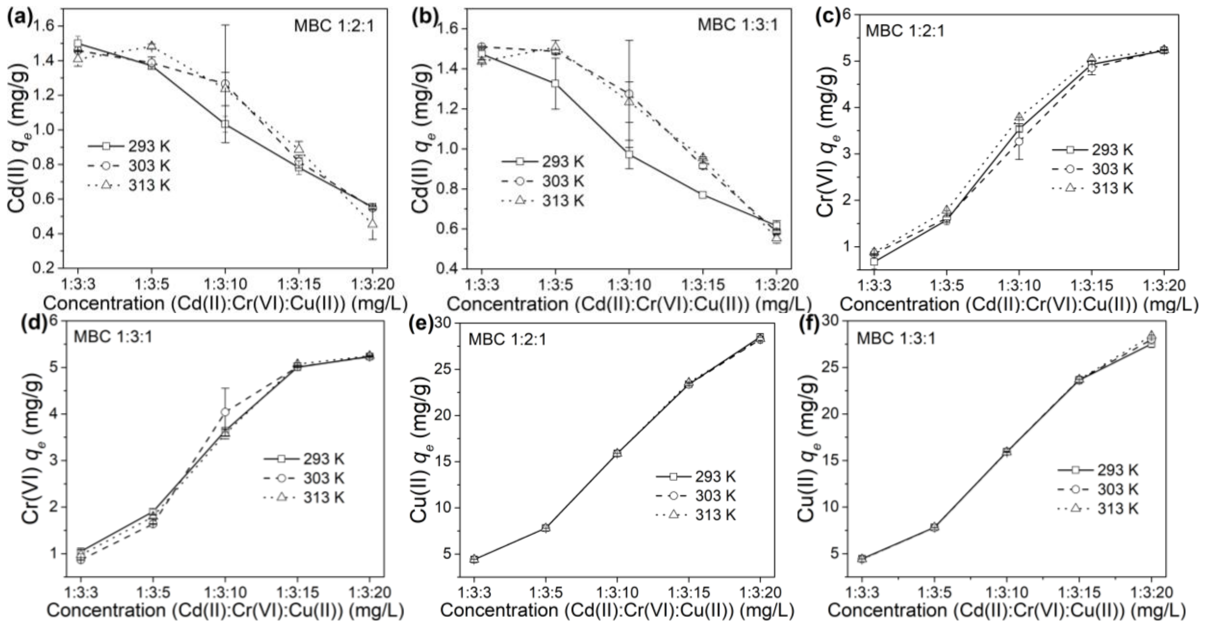


SI Fig. 4 Effect of temperature on adsorption of (a) Cd(II) on MBC 1:2:1,(b) Cd(II) on MBC 1:3:1; (c) Cr(VI) on MBC 1:2:1 (d) Cr(VI) on MBC 1:3:1; (e) Cu(II) on MBC 1:2:1, (f) Cu(II) on MBC 1:3:1

SI Table 1. Ionic properties of Cd(II), Cr(VI) and Cu(II)

| Property | Cr(VI) | Cu(II) | Cd(II) |
|---|---|---|---|
| Atomic weight | 51.01 | 63.55 | 112.41 |
| Molecular weight | 294.18 | 134.45 | 769.52 |
| Electronic configuration | $[Ar]3d^5 4s^1$ | $[Ar]3d^{10}4s^1$ | $[Kr]4d^{10}5s^2$ |
| Electronegativity | 1.66 | 1.9 | 1.69 |
| Ionic radii ($A^0$) | 0.52 | 0.72 | 0.95 |
| Hydrated radii ($A^0$) | 4.61 | 4.19 | 4.26 |
| Coordination number | 6 & 4 | 2 & 4 | 6 & 4 |
| Standard Reduction Pot. (V) | $Cr^{6+} + 3e^- \rightarrow Cr^{3+}$(1.1)<br>$Cr^{3+}3e^- \rightarrow Cr$ (-0.74) | $Cu^{2+} + 2e^- \rightarrow Cu$ (0.34) | $Cd^{2+} + 2e^- \rightarrow Cd$ (-0.403) |

Source: [1, 2]

SI Table 2. Interactions of metal ions in ternary systems

| Metal System | MBC 1:2:1 | | MBC 1:3:1 | |
|---|---|---|---|---|
| | *$R_i$ (%)* | Interaction | *$R_i$ (%)* | Interaction |
| Cadmium | | | | |
| Cd(II) + Cr(VI) + Cu(II) | 103 | No interaction | 81 | Antagonistic |
| Chromium | | | | |
| Cr(VI) + Cd(II) + Cu(II) | 1510 | Synergistic | 29298 | Synergistic |
| Copper | | | | |
| Cu(II) + Cd(II) + Cr(VI) | 109 | Slightly synergistic | 111 | Slightly synergistic |

SI Table 3. Cost analysis of MBC 1:2:1/MBC 1:3:1 production

| Preparation phase | | Cost assignment | Cost for ($) | | Cost in stages ($) | Cost MBC 1:2:1/MBC 1:3:1 ($) |
|---|---|---|---|---|---|---|
| | | | Chemicals | Energy | | |
| Arrangement of precursor for | Procurement (FLC) | $Cost_{AAP}$ | 0.125/0.1 | – | 0.365/0.34 | 5.5627/5.5154 |
| | Transport | | – | 0.23 | | |
| | Packaging and storage | | 0.01 | 0.01 | | |
| Pre-treatment of precursors | Cleaning | $Cost_{PP}$ | 0.18/0.2 | – | 1.48/1.5 | |
| | Drying | | – | 0.4 | | |
| | Size reduction | | – | 0.9 | | |
| Preparation and pyrolysis | Heating | $Cost_{Pyro}$ | – | 0.87 | 1.591/1.559 | |
| | $FeCl_3$ | | 0.15/0.12 | | | |
| | $FeSO_4 \cdot 7H_2O$ | | 0.014/0.012 | | | |
| | Shaken | | | 0.08 | | |
| | Centrifugation | | | 0.027 | | |
| | Washing | | 0.45 | | | |
| Chemical activation | NaOH treatment | $Cost_{CA}$ | 1.6 | | 1.615 | |
| | Stirring | | | 0.015 | | |
| Others cost | Offset cost | $Cost_{Others}$ | 10% of $ | | 0.5057/0.5014 | |

Sources: [3, 4]

## REFERENCES


1. Jain, M., et al., *Adsorption of heavy metals from multi-metal aqueous solution by sunflower plant biomass-based carbons.* International Journal of Environmental Science and Technology, 2015. **13**(2): p. 493-500.
2. Khelifi, O., et al., *Response Surface Modeling and Optimization of Ni(II) and Cu(II) Ions Competitive Adsorption Capacity by Sewage Sludge Activated Carbon.* Arabian Journal for Science and Engineering, 2021. **47**(5): p. 5797-5809.
3. Das, T., A. Debnath, and M.S. Manna, *Adsorption of malachite green by Aegle marmelos-derived activated biochar: Novelty assessment through phytotoxicity tests and economic analysis.* Journal of the Indian Chemical Society, 2024. **101**(9): p. 101219.
4. Unuabonah, E.I., et al., *Successful scale-up performance of a novel papaya-clay combo adsorbent: up-flow adsorption of a basic dye.* Desalination and Water Treatment, 2015. **56**(2): p. 536-551.
